# Impact of controlled high-sucrose and high-fat diets on eosinophil recruitment and cytokine content in allergen-challenged mice

**DOI:** 10.1371/journal.pone.0255997

**Published:** 2021-08-12

**Authors:** Caroline M. Percopo, Morgan McCullough, Ajinkya R. Limkar, Kirk M. Druey, Helene F. Rosenberg

**Affiliations:** 1 Inflammation Immunobiology Section, Laboratory of Allergic Diseases, National Institute of Allergy and Infectious Diseases, National Institutes of Health, Bethesda, Maryland, United States of America; 2 Lung and Vascular Inflammation Section, Laboratory of Allergic Diseases, National Institute of Allergy and Infectious Diseases, National Institutes of Health, Bethesda, Maryland, United States of America; Universidade Federal do Rio de Janeiro, BRAZIL

## Abstract

Despite an ongoing focus on the role of diet in health and disease, we have only a limited understanding of these concepts at the cellular and molecular levels. While obesity has been clearly recognized as contributing to metabolic syndrome and the pathogenesis of adult asthma, recent evidence has linked high sugar intake alone to an increased risk of developing asthma in childhood. In this study, we examined the impact of diet in a mouse model of allergic airways inflammation with a specific focus on eosinophils. As anticipated, male C57BL/6 mice gained weight on a high-calorie, high-fat diet. However, mice also gained weight on an isocaloric high-sucrose diet. Elevated levels of leptin were detected in the serum and airways of mice maintained on the high-fat, but not the high-sucrose diets. We found that diet alone had no impact on eosinophil numbers in the airways at baseline or their recruitment in response to allergen (*Alternaria alternata*) challenge in either wild-type or leptin-deficient ob/ob mice. However, both bronchoalveolar lavage fluid and eosinophils isolated from lung tissue of allergen-challenged mice exhibited profound diet-dependent differences in cytokine content. Similarly, while all wild-type mice responded to allergen challenge with significant increases in methacholine-dependent total airway resistance (R_rs_), airway resistance in mice maintained on the isocaloric high-sucrose (but not the high-calorie/high-fat) diet significantly exceeded that of mice maintained on the basic diet. In summary, our findings revealed that mice maintained on an isocaloric high-sucrose diet responded to allergen challenge with significant changes in both BAL and eosinophil cytokine content together with significant increases in R_rs_. These results provide a model for further exploration of the unique risks associated with a high-sugar diet and its impact on allergen-associated respiratory dysfunction.

## Introduction

Despite ongoing focus on the role of diet in promoting health and longevity, we have only a limited understanding of the nature and impact of concepts at the cellular and molecular levels. While obesity (defined as a body mass index [BMI] > 30) has been identified as a hyperinflammatory state that contributes to metabolic syndrome (a cluster of findings that includes hypertension, elevated plasma glucose levels, and abnormal cholesterol levels [1]), the global impact of an unhealthy diet on immune and inflammatory responses remains poorly understood. For example, while obesity has been associated with a distinct asthma phenotype [2–4], excessive sugar intake alone has been linked to an increased risk of developing childhood asthma [5,6].

The role of diet promoting allergic airway dysfunction has been explored in mice that are genetically obese (i.e., ob/ob mice) and in wild-type mice that have gained excessive weight on high-fat diets [7–10]. Similarly, Singh *et al*. [11] and Leishangtham *et al*. [12] reported that mice maintained on diets that were high in fructose did not become obese, but did develop dysregulated airway responses both at baseline and in response to allergen challenge.

In this study, we focused on the impact of diet on inflammatory responses in the airways with a specific focus on eosinophils. While eosinophils have been implicated in energy homeostasis and regulation of adipose tissue, the underlying mechanisms and associated outcomes remain controversial [13–18]. Recent studies documenting eosinophil heterogeneity have emphasized the diversity and functional versatility of this leukocyte subpopulation and have focused on the influence of both environment and location on the eosinophil phenotype [19–25]. Collectively, these findings represent an important extension of the LIAR hypothesis [26] as they suggest that eosinophils not only promote local immunomodulation, they may also be influenced by specific conditions in the microenvironment. For example, Reichman *et al*. [19] identified unique patterns of gene expression when comparing tumor eosinophils to those isolated from normal colon tissue. Similarly, a recent study by Sek *et al*. [20] revealed that eosinophils isolated from muscle tissue of mdx (muscular dystrophy) mice differentially expressed tissue remodeling genes and the immunomodulatory receptor, *Trem2*. Likewise, Ma *et al*. [25] documented 100-fold differences in IL-16 content in peripheral blood eosinophils from healthy donors at levels that varied directly with donor BMI.

Here, we examined the impact of diet at baseline and in response to allergen-challenge. Specifically, we evaluated eosinophil recruitment, eosinophil and bronchoalveolar lavage (BAL) fluid cytokine content, and airway hyperresponsiveness in allergen-challenged mice that were maintained on defined basic, high-sucrose, or high-fat diets. We were particularly intrigued to find that, although diet had no apparent impact on eosinophil recruitment in response ot allergen challenge, both BAL and eosinophil cytokine content differed significantly under these conditions.

## Materials and methods

### Mice and diets

Male C57BL/6 mice were obtained from The Jackson Laboratory (stock no. 000664). Mice were initially maintained on standard mouse lab chow (Formulation 5010; LabDiet, Land O’Lakes, Inc.). At six weeks of age, mice were switched from standard chow to one of three specific formulated diets (Research Diets, New Brunswick NJ). Diets used in this study included D12450J (basic; 10% fat low-sucrose), D12450B (high-sucrose; 10% fat, high-sucrose) and D12492 (high-fat; 60% fat, low-sucrose). A description of each diet, including amounts of sucrose per 100 g and caloric content can be found in **S1 Table**. Mice were housed at 5 per cage in a room with a standard dark/light cycle (12 hr/12 hr) and provided with drinking water *ad libitum*. For some studies, we obtained C57BL/6 male mice from the Jackson Laboratory that were started on the aforementioned high-fat or high-sucrose diets as above (stock nos. 380056 and 380050, respectively) beginning at age six weeks; these diets were continued once these mice arrived in our vivarium. Leptin-deficient ob/ob mice (male, C57BL/6 background) were obtained from the Jackson Laboratory (stock no. 000632) and were maintained on each of the three aforementioned diets. Weights of all mice were recorded weekly. Serum samples were collected from each mouse every 2–3 weeks. All mice were maintained under pathogen-free conditions at an American Association for the Accreditation of Laboratory Animal Care accredited animal facility at the NIAID and housed in accordance with the procedures outlined in the Guide for the Care and Use of Laboratory Animals under an animal study proposal approved by the NIAID Animal Care and Use Committee. All mice were used at ages indicated in each experiment.

### Ethics statement

*In vivo* efficacy studies were approved by the NIAID Institutional Animal Care and Use Committee. Animal work was conducted adhering to the institution’s guidelines for animal use and followed the guidelines and basic principles in the United States Public Health Service Policy on Humane Care and Use of Laboratory Animals, and the Guide for the Care and Use of Laboratory Animals by certified staff in an Association for Assessment and Accreditation of Laboratory Animal Care (AAALAC) International accredited facility

### Intranasal challenge with *Alternaria alternata (Aa)*

In some experiments, mice maintained for 10 weeks on each of the aforementioned diets were challenged intranasally on days 0, 3, and 6 with 50 μg of a filtrate of *Alternaria alternata* (*Aa*; Stallergenes Greer, Lenoir, NC) in a volume of 50 μL as previously described [27,28]. Mice were evaluated on day 10 thereafter. BAL fluid was collected as described below for the evaluation of airway cytokines, leukocyte totals, and leukocyte differential counts. Single-cell suspensions were prepared from whole lung tissue for analysis using flow cytometry and for collection of eosinophils by fluorescence-activated single-cell sorting (FACS) as described below.

### Bronchial alveolar lavage (BAL)

Mice were sacrificed by isoflurane inhalation after at least 10 weeks on specific diets at time points as indicated. BAL fluid was obtained via two sequential intratracheal infusions of 0.8 mL of phosphate-buffered saline (PBS) with 0.1% bovine serum albumin (BSA); approximately 1 mL of BAL fluid was recovered from each mouse. Cytospin slides prepared for each sample were stained with modified Giemsa (Diff-Quik) and evaluated by visual inspection under light microscopy (64x magnification).

### Preparation of single-cell suspensions from whole lung tissue

Preparation of single-cell suspensions from whole lung tissue was performed as described in our previous publications [27,29]. Briefly, mice were sacrificed by isoflurane inhalation and perfused with 0.8 mL of 10 μM EDTA in PBS. Lung tissue was digested in a total of 9 mL of digestion medium (20 μg/mL DNase, 2 mg/mL Collagenase D, and 5% fetal bovine serum [FBS] in RPMI 1640 medium) at 37°C on a turning wheel for 90 min, followed by addition of 180 μL of 0.5M cold EDTA and a 5-minute incubation at room temperature. The sample was then filtered (70-micron filter) followed by the addition of 10 mL cold Würzburg buffer (PBS with 0.3% BSA, 5 mM EDTA) with 4 μg/mL DNase I. The digested cells were pelleted at 300 *x g* for 5 minutes followed by resuspension in 2 mL of ACK lysis buffer. Lysis was halted with an additional 10 mL of Würzburg buffer. The cells were then pelleted again and resuspended in 10 ml of PBS with 0.1% BSA. Cells were counted on a LUNA-FL automated fluorescence cell counter and viability was determined.

### Flow cytometric analysis and FACS-isolation of lung eosinophils

Flow cytometry and FACS were performed as described previously [27,29]. Briefly, cells isolated from lung tissue as described above were pelleted and resuspended at 10^7^ cells/mL in PBS with 0.1% BSA. Non-specific antibody binding was blocked with anti-CD16/CD32 followed by a master mix antibody cocktail (50 μL per 10^7^ cells) that included 10 μL of each of the following antibodies: anti-CD45-eF450 (clone 30-F11; eBioscience), anti-CD11c-AF700 (clone N418; eBioscience), anti-Gr1 (Ly-6G and Ly-6C)-APC (clone RB6-8C5; BD Biosciences), anti-Siglec F-PE (clone E50-2440; BD Biosciences) and anti-MHCII (I-A/I-E)-PE-CY-7 (clone M5/114.15.2; eBioscience). Cells were incubated with antibodies at 4°C for 30 min while protected from light, then pelleted and resuspended in PBS with 0.5% BSA at 10^8^ cells/mL. Samples were evaluated and sorted using a FACSAria Fusion cytometer (BD Biosciences). Eosinophils were identified as CD45^+^/CD11c^-^/GR1^lo^/MHCII^-^/Siglec F^+^ cells. Additional details are provided in S1 Appendix.

### Preparation of lysates from FACS-isolated eosinophils

FACS-isolated eosinophils were pelleted at 300 *x g* at 4°C and washed twice in PBS. After washing, the pelleted eosinophils were resuspended in lysis buffer (1% Igepal CA-630 in 20 mM Tris-HCl (pH 8.0), 137 mM NaCl, 10% glycerol, 2 mM EDTA, with protease inhibitor cocktail at 10^7^ cells/mL followed by incubation with end-over-end rotation at 4°C for 30 min. Cell debris was removed by centrifugation at 14,000 *x g* for 10 minutes at 4°C. The lysate was separated from cell debris and stored at -20°C until use. Protein concentration in each lysate was determined by BCA assay (Pierce).

### Cytokine profiling

Profiling of BAL fluid and eosinophil lysates was performed using the Mouse Cytokine Array Panel A (R & D Systems, Minneapolis, MN) as per manufacturer’s instructions and as previously described [20,28]. Briefly, combined samples (from n = 5 mice) were incubated with the Mouse Cytokine Array Panel A detection antibody cocktail for one hour at room temperature. BAL fluid or lysate samples were then added to pre-wetted membranes embedded with capture antibodies. The sample and membrane were incubated overnight at 4°C on a rocking platform. Membranes were washed as per the manufacturer’s instructions and then incubated for 30 minutes with IRDye 800CW Streptavidin (LI-COR Biosciences, Lincoln, NE) at room temperature on a rocking platform that was protected from light; this was followed by a final washing step. Data were collected using an Odyssey CLx imaging system (LI-COR) and analyzed using a grid array. Additional details are provided in S1 Appendix.

### ELISAs

Concentrations of IL-16 and IL-1Ra in BAL fluid and leptin in serum and BAL fluid were determined using Duo-Set ELISAs (R & D Systems) as per the manufacturer’s instructions.

### Airway resistance in response to methacholine

The flexiVent FX (SCIREQ) system and its accompanying software were used to assess respiratory mechanics as per the manufacturer’s instructions. Each mouse was weighed before anesthetizing with ketamine/xylazine. A tracheostomy was performed and a 19-gauge cannula was inserted and secured with a 4–0 silk suture. Each mouse was connected to flexiVent to begin ventilation while vecuronium was administered. After a 5-minute wait time, deep inflation was performed to check for potential leaks. Responses to increasing concentration of methacholine (0, 3.125, 6.25, 12.5, and 25 mg/mL) were examined using 12 repeats in two separate trials for each dose. Total resistance (R_rs_) was recorded for each mouse and at each dose of methacholine. Additional details are provided in S1 Appendix.

### Statistical methods

All data were evaluated using algorithms within GraphPad Prism version 9.2. Statistical evaluations included Student’s t-tests, one-sample t-tests, Mann-Whitney U-tests, and one-way and two-way ANOVA with post-hoc tests. The specific test used and number of mice per group are as indicated for each set of experiments in the Figure Legends.

## Results

### Cytokine profiling and leukocyte recruitment to the airways of mice maintained on high-sucrose or high-fat diets

**Fig 1:**
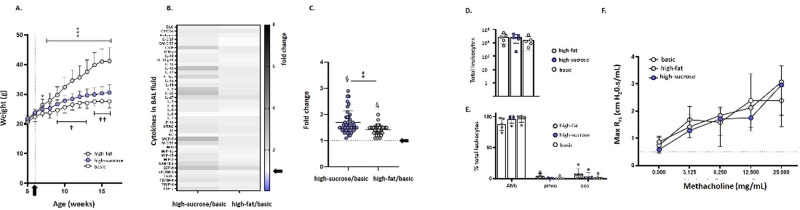
An isocaloric high-sucrose diet results in weight gain and modest elevations in proinflammatory cytokines in the airways. **(A)** Male C57BL/6 mice were maintained on basic (10% fat, 3.82 kcal/g), isocaloric/high sucrose (10% fat; 3.82 kcal/g with 33.6 g sucrose/g), or high-caloric/high-fat (60% fat, 5.21 kcal/g) diets (from Research Diets, Inc.; see S1 Table for full details) for 10 weeks, beginning at 6 weeks of age (at the arrow and vertical dotted line). Body weights (± standard deviation [SD], in grams) are as indicated; n = 18–20 mice per group, *p < 0.05, ***p < 0.001, high-fat *vs*. basic diet; ^†^*p* < 0.05, ^††^*p* < 0.01, high-sucrose *vs*. basic diet, 2-way ANOVA with Dunnett’s post hoc test. **(B)** Heatmap and (**C**) semiquantitative analysis of cytokine profiles in bronchoalveolar lavage (BAL) fluid. The data shown are ratios of signals detected in BAL fluid of mice maintained on a high-sucrose or high-fat diets, each normalized to signals from BAL fluid of mice maintained on the basic diet and presented as fold-increase, n = 5 mice as described in the Methods. Arrows indicate no increase over signal detected in mice on basic diet (fold change = 1.0). Mean and SD for all cytokines are as shown in (C) at 1.7 ± 0.44 (high-sucrose/basic) and 1.4 ± 0.18 (high fat/basic); **p < 0.01 (high-sucrose/basic *vs*. high-fat/basic, Mann-Whitney U-test); ^§^*p*< 0.001 *vs*. theoretical mean (= 1.0), one-sample t-tests. **(D)** Total leukocytes and **(E)** leukocyte differentials (% alveolar macrophages [AMs], neutrophils [pmns], and eosinophils [eos]) in BAL fluid of mice maintained on high-sucrose, high-fat, and basic diets. (**F**) Airway resistance (R_rs_; cmH_2_O.s/mL) observed in response to increasing concentrations of methacholine (0–25 mg/mL) in mice maintained on basic, high-sucrose, or high-fat diets (n = 5 mice per group). No statistically significant differences were identified.

C57BL/6 male mice maintained on a standard mouse diet (see Methods) were weighed at baseline (at 5 and 6 weeks of age) before they were shifted to one of the three defined diets (see **S1 Table**). As shown in **Fig 1A**, the mice on the high-fat diet gained significantly more weight overall than did mice on the high-sucrose or basic diets. Significant increases in body weight were observed as early as week 7. Weight gain continued at a rate of ~ 2.0 g/week until leveling off at weeks 15–16. Although not as dramatic as the weight gain experienced by mice on the high calorie/high-fat diet, mice maintained on a high-sucrose diet (which was isocaloric with the basic diet, see S1 Table) gained more weight over time than did the mice maintained on the basic diet (0.82 *versus* 0.68 g/week, respectively). Mice maintained on the high-sucrose diet were significantly heavier than those on the basic diet as early as week 9, and remained consistently heavier throughout the experimental period as shown.

Mice in this experiment underwent no additional manipulations and were sacrificed at week 16 for further evaluation. Rather than focusing on single mediators, we performed a global assessment of cytokines in the airways using a membrane-based array method that detects soluble mediators via a “sandwich” method analogous to that used by a standard ELISA. This method was featured in several earlier studies [20,25,28] including one in which we validated array findings in lysates of peripheral blood eosinophils isolated from ~40 independent donors with standard cytokine-specific ELISAs [25].

Individual signals for each cytokine obtained from BAL fluid samples of mice maintained for 10 weeks on either high-fat or high-sucrose diets were normalized as previously described [25] to facilitate intergroup comparisons. Our findings revealed modest cytokine enrichment in BAL fluid samples from mice that were maintained on both high-sucrose and high-fat diets compared to the basic diet. The average increases for all 40 cytokines evaluated were 1.7 ± 0.44-fold and 1.4 ± 0.18-fold in BAL fluid from mice on the high-sucrose and high-fat diets, respectively (**Fig 1B** and **1C**). Collectively, these results suggest that (1) prolonged intake of high-fat or high-sucrose diets promotes modest increases in airway cytokines even in the absence of allergic provocation, and (2) these responses appear to be diet-related and do not correlate with the extent of weight gain. Interestingly, the observed increases in BAL cytokines in otherwise unmanipulated mice had no impact on total airway leukocytes (**Fig 1D**), the leukocyte differential (**Fig 1E**), or on airway responses to methacholine challenge at baseline (i.e., no allergen challenge; **Fig 1F**).

### Cytokine profiling and leukocyte recruitment to the airways in mice maintained on high-fat or high-sucrose diets and subjected to allergen challenge

**Fig 2:**
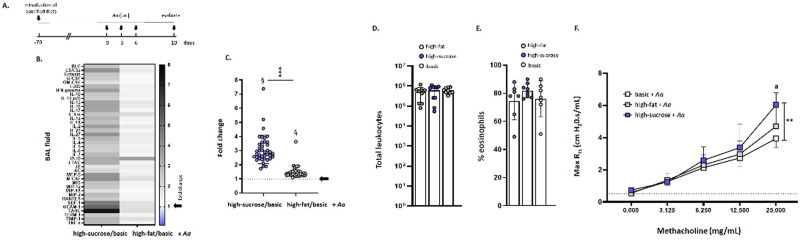
Cytokine and airway responses of wild-type mice maintained high-sucrose, high-fat, or basic diets after challenge with *Alternaria alternata*. **(A)** Protocol used for allergen challenge. After 70 days (10 weeks) on specified diets, mice were challenged intranasally with a filtrate of *A*. *alternata* (*Aa*; 50 μg/mouse in 50 μL PBS) on days 0, 3, and 6 and evaluated on day 10 as previously described [27,28]. **(B)** Heatmap and (**C**) semiquantitative analysis of cytokine profiles detected in BAL fluid after repetitive challenge with *Aa* as in (A). The data shown are ratios of signals detected in BAL fluid from *Aa*-challenged mice maintained on high-sucrose or high-fat diets, each normalized to signals from BAL fluid from *Aa*-challenged mice maintained on the basic diet and presented fold-increase, n = 5 mice as described in the Methods. Arrows indicate no increase over signal detected in mice on basic diet (fold change = 1.0). Mean and SD for all cytokines are as shown in (C) at 3.1 ± 1.0 (high-sucrose/basic) and 1.5 ± 0.41 (high fat/basic); ***p < 0.001 (high-sucrose/basic *vs*. high-fat/basic, Mann-Whitney U-test); ^§^*p*< 0.001 *vs*. theoretical mean (= 1.0), one-sample t-tests. See S1 Fig for additional information. **(D)** Total leukocytes and **(E)** % eosinophils detected in BAL fluid of *Aa*-challenged mice maintained on high-fat, high-sucrose, or basic diets. **(F)** Total airway resistance (R_rs_; cmH_2_O.s/mL) observed in response to increasing concentrations of methacholine (0–25 mg/mL) in mice maintained on basic, high-fat, or high-sucrose diets followed by *Aa* challenge as (A), n = 5 mice per group; ^a^statistically significant increases in R_rs_ detected in *Aa*-challenged mice over levels observed their respective baselines as shown in Fig 1F; **p < 0.01 high-sucrose + *Aa vs*. basic + *Aa*, 2-way ANOVA with Tukey’s post hoc test.

Mice maintained on high-fat, high-sucrose, or basic diets for 10 weeks (from day -70 to day 0 as shown in **Fig 2A**) were challenged intranasally with a filtrate of the fungal allergen, *A*. *alternata* (*Aa*; 50 μg per inoculation) on days 0, 3, and 6, followed by evaluation on day 10. This allergen challenge model has been used previously to examine airway inflammation and eosinophil recruitment [27,28,30]. As shown previously [31], intranasal challenge with *Aa* results in eosinophil recruitment and increases in airway cytokines in mice maintained on a standard laboratory diet.

Cytokine profiling was performed on BAL fluid collected from these mice. Signals for cytokines detected in BAL fluid from *Aa*-challenged mice maintained on high-sucrose and high-fat diets were normalized to those from *Aa*-challenged mice that were maintained on the basic diet. Similar to what we observed in mice that had not undergone allergen challenge (Fig 1B and 1C) we detected comparatively higher cytokine levels overall in BAL fluid from mice maintained on the high-sucrose and high-fat diets (**Fig 2B** and **2C**). However, in this case, the BAL fluid from allergen-challenged mice maintained on the high-sucrose diet was notably cytokine-enriched compared to samples from mice maintained on a basic diet. As shown in Fig 2C, the average fold changes were 3.1 ± 1.0-fold and 1.5 ± 0.41-fold for mice on high-sucrose and high-fat diets, respectively. Fold-change calculations for individual cytokines are shown in **S1 Fig**. As anticipated, allergen challenge elicited leukocyte recruitment to the airways (**Fig 2D**) including predominantly eosinophils (70–80%; **Fig 2E**) with no significant differences detected between the three groups. Interestingly, total airway resistance (R_rs_) was significantly higher in allergen-challenged mice that had been maintained on a high-sucrose diet compared to those maintained on the basic diet (**Fig 2F**).

### Impact of leptin on airway inflammation and cytokine levels

**Fig 3:**
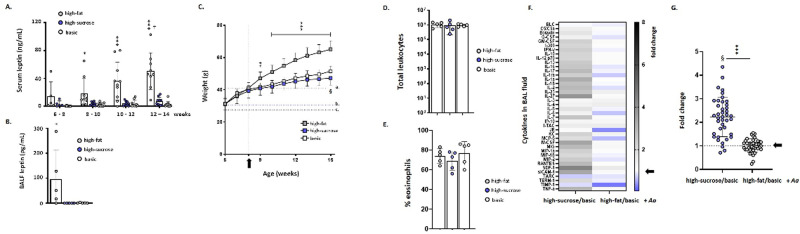
Serum leptin levels have no impact on eosinophil recruitment or cytokine responses in mice maintained on a high-sucrose diet. **(A)** Serum leptin levels over time in C57BL/6 male mice maintained on basic, high-fat, or high-sucrose diets; n = 5–10 mice per group, *p < 0.05, ***p < 0.001 for mice on high-fat diet *vs*. basic or high-sucrose diets, ^†^*p* < 0.05 for mice maintained on a high-fat diet at weeks 12–14 *vs*. week 6–8; 2 –way ANOVA with Tukey’s post hoc test; see also S2 Fig. (**B**) Leptin levels in BAL fluid of 16-week-old mice after 10 weeks on each diet (n = 5 mice per group, *p < 0.05 *vs*. mice maintained on basic or high-sucrose diets, 1-way ANOVA). **(C)** Weights of male leptin-deficient ob/ob mice on the C57BL/6 background maintained on high-fat, high-sucrose, or basic diets from 8 weeks (at the arrow and vertical dotted line) through 15 weeks of age; n = 10–15 mice per group, **p < 0.01, ***p < 0.001, high-fat *vs*. basic diet, ^§^*p* < 0.05, basic *vs*. high-sucrose diet, 2 –way ANOVA with Tukey’s post-hoc test. Dotted horizontal lines a., b., and c. indicate peak weights of wild-type male C57BL/6 mice maintained on high-fat, high-sucrose, and basic diets, respectively as shown in Fig 1A. **(D)** Total leukocytes and **(E)** % eosinophils detected in BAL fluid of *Aa*-challenged ob/ob mice maintained on high-fat, high-sucrose, or basic diets. **(F)** Heatmap and (**G**) semiquantitative analysis of cytokine profiles detected in BAL fluid of ob/ob mice after repetitive challenge with *Aa* as shown in Fig 2A. The data shown are ratios of signals detected in BAL fluid from *Aa*-challenged ob/ob mice maintained on high-fat or high-sucrose diets, with each normalized to signals from BAL fluid from *Aa*-challenged ob/ob mice maintained on the basic diet and presented fold-increase, n = 5 mice per group as described in the Methods. Arrows indicate no increase over signal detected in mice on basic diet (fold change = 1.0). Mean and SD for all cytokines are as shown in (G) at 2.2 ± 0.84 (high-sucrose/basic) and 1.0 ± 0.28 (high-fat/basic); ***p < 0.001 (high-sucrose/basic *vs*. high-fat/basic, Mann-Whitney U-test); ^§^*p*< 0.001 *vs*. theoretical mean (= 1.0), one-sample t-tests.

Leptin is a critical regulator of energy balance and satiety via its actions within the hypothalamus and has distinct roles in immune cell activation and the control of inflammatory pathology [32,33]. Conus *et al*. [34] identified leptin as a pro-survival factor for human eosinophils, and Liu *et al*. [35] reported that leptin modulated eosinophil survival, activation, and migration responses in a traditional ovalbumin sensitization and challenge model of allergic airways inflammation in obese mice. We first examined serum concentrations of leptin over time in mice maintained on each of the three defined diets. Although mice on the high-sucrose diet gained more weight over time than did the mice on the basic diet (see Fig 1A), significant increases in serum (**Fig 3A**) and BAL leptin (**Fig 3B**) over baseline levels and over time were detected in mice on the high-fat diet only. Interestingly, while serum leptin levels correlated directly with body weight in mice on the high-fat and basic diets, no correlation between serum leptin levels and body weight was observed in mice maintained on the high-sucrose diet (**S2 Fig**).

We examined the responses of leptin-deficient (ob/ob) mice to each of the three defined diets both at baseline and in response to allergen challenge. Absolute leptin deficiency was confirmed in all mice in this study. As anticipated, ob/ob mice maintained on the basic diet gained weight over time (2.0 g/week) at a rate similar to that observed for wild-type mice maintained on a high-fat diet. This weight gain was exceeded by that of ob/ob mice maintained on the high-fat diet (3.8 g/week; **Fig 3C**). Interestingly, ob/ob mice on the high-sucrose diet gained weight in a pattern that was similar to their ob/ob counterparts maintained on the defined basic diet. The role played by leptin on the differential weight gain observed in Fig 1A (i.e., mice on high-sucrose *versus* defined basic diets) remains to be determined.

**Fig 4:**
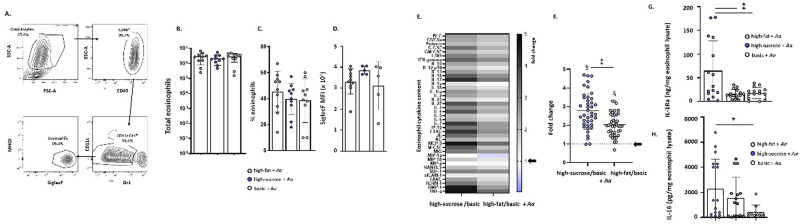
Cytokine-enriched eosinophils isolated from the lungs of *Aa*-challenged mice maintained on a high-sucrose diet. Mice were maintained on specific diets and challenged with *Aa* as shown in Fig 2A. (**A**) Gating strategy used to isolate eosinophils from mouse lung tissue. As shown, FACS-isolated eosinophils are live, high forward/side scatter (granulocyte gate), CD45^+^CD11c^-^Gr1^lo^MHCII^-^SiglecF^+^ cells. **(B)** Total eosinophils, **(C)** percent eosinophils, and **(D)** eosinophil Siglec F MFI identified in mouse lung tissues. (**E**) Heatmap and (**F**) semiquantitative analysis of the cytokine contents of FACS-isolated eosinophils from lung tissue of *Aa*-challenged mice. The data shown are ratios of the cytokine contents of eosinophils isolated from lung tissue of *Aa*-challenged mice maintained on high-fat or high-sucrose diets normalized to signals obtained from eosinophils isolated from *Aa*-challenged mice maintained on the basic diet, n = 5 mice per group as described in the Methods. Arrows indicate no increase over signal detected in mice maintained on the basic diet (fold change = 1.0). Mean and SD for all cytokines are as shown in (F) at 2.8 ± 0.98 (high-sucrose/basic) and 2.0 ± 0.63 (high-fat/basic); **p < 0.01 (high-sucrose/basic *vs*. high-fat/basic, Mann-Whitney U-test; ^§^*p*< 0.001 *vs*. theoretical mean (= 1.0), one-sample t-tests. See S3 Fig for additional information. (**G)** IL-1Ra (ng/mg lysate) and (**H**) IL-16 (pg/mg lysate) of eosinophils isolated from *Aa*-challenged mice on high-sucrose, high-fat, or basic diets (*p < 0.05, **p < 0.01 between groups as shown, 1-way ANOVA with Tukey’s post hoc test).

Ob/ob mice maintained on each of the three diets were challenged with allergen as shown in Fig 2A. All ob/ob mice responded to allergen challenge with leukocyte recruitment (**Fig 3D**) including 70–80% eosinophils (**Fig 3E**). Analogous to findings with wild-type mice, diet had no impact on total leukocyte or eosinophil recruitment. However, and similar (but not identical) to findings shown in Fig 2B for wild-type mice, we detected significantly higher cytokine levels overall in BAL fluid from allergen-challenged ob/ob mice maintained on the high-sucrose (but not the high-fat) diet (**Fig 4F** and **4G**). The average increases over signals detected at baseline (i.e., in BAL fluid from *Aa*-challenged ob/ob mice maintained on the defined basic diet) were 2.2 ± 0.84-fold *vs*. 1.0 ± 0.28-fold, for *Aa*-challenged mice on high-sucrose *versus* high-fat diets, respectively. Fold-change calculations for individual cytokines are shown in **S3 Fig.**

### Eosinophil cytokine content

Eosinophils have been identified as critical mediators of airway hyperresponsiveness in allergen challenge models [36–38]. Although diet had no apparent impact on eosinophil recruitment to the airways, we performed experiments that would permit us to identify differences in eosinophil cytokine content. Several previous studies focused on eosinophil heterogeneity and the influence of the tissue microenvironment on eosinophil structure and contents [19,20]. Mice on defined diets (basic, high-fat, or high-sucrose) were challenged with *Aa* as shown in Fig 2A and eosinophils in lung tissue (live CD45^+^CD11c^-^Gr1^lo^MHCII^-^SiglecF^+^ were isolated by FACS (**Fig 4A**). As anticipated, diet had no impact on the total (**Fig 4B**) or percentage of eosinophils (**Fig 4C**) isolated from whole lung tissue. Diet also had no impact on the mean fluorescence intensity (MFI) of Siglec F (**Fig 4D**), a sialic acid-binding immunoglobulin-type lectin and cell surface marker of eosinophil activation and transit to the lung [39,40].

Lysates were prepared from isolated eosinophils and their cytokine contents were examined by profiling as described above. As shown in **Fig 4E** and **4F**, eosinophils isolated from mice maintained on the high-sucrose and high-fat diets were cytokine-enriched compared to those isolated from allergen-challenged mice maintained on a basic diet. Increases in eosinophil cytokine content over baseline (i.e., cytokine content in eosinophils isolated from *Aa*-challenged mice on the defined basic diet) were identified as 2.8 ± 0.98-fold *vs*. 2.0 ± 0.63-fold for mice high-sucrose *vs*. high-fat diets, respectively. Among these, differences in critical mediators reproduced by ELISA included eosinophil IL-1Ra ([41]; **Fig 5G**) and IL-16 ([25,42]; **Fig 5H**), which are the two mediators with the highest coefficient of variation in human eosinophils (see Table 3 and Fig 2 of reference [25]).

## Discussion

Sugar in the form of glucose (dextrose), fructose, and sucrose (a dimer of glucose and fructose) added to pre-sweetened beverages and processed food are a mainstay of the typical American diet. While current U. S. Food and Drug Administration guidelines recommend a daily limit of ≤50 grams of added sugar (i.e., <10% of total calories), adults and children in the U.S. consume on average 77 and 81 grams of added sugar per day, respectively [43]. While obesity is recognized as a primary contributor to the condition known as the metabolic syndrome (a constellation of findings associated with an increased risk of cardiovascular disease and type 2 diabetes), epidemiologic studies have highlighted high sugar intake as an independent risk factor associated with the development of childhood asthma [5,6]. In response to this ongoing concern, a 2017 statement from the American Academy of Pediatrics advocated limits on the intake of fruit juices in children under the age of five and noted that these beverages might be avoided completely in infants who are less than one year of age [44].

In this study, we detected comparatively higher cytokine levels in the airways of wild-type mice maintained on the high-sucrose and high-fat diets. BAL fluid from allergen-challenged wild-type mice maintained on the high-sucrose diet was notably cytokine-enriched. Allergen-challenged mice maintained on the high-sucrose diet also exhibited profound increases in total airway resistance that were significantly larger than those of mice on either the high-fat or the basic diets. Of note, these experiments focused on the overall increase in inflammatory cytokines and were not designed to characterize the actions of any single mediator. Indeed, given the extensive network that has been attributed to cytokine interactions [45], this may not even be fully feasible. Future studies might also examine the roles of eicosanoid mediators, including leukotrienes, which have been identified as among the critical mediators in some asthmatic patients [46].

We also examined the impact of diet on total airway resistance and eosinophil recruitment in a characterized model of allergic asthma. Eosinophils have been identified as significant mediators of allergic airway responses in both human subjects and mouse models [36–38,47,48]. Although the LIAR hypothesis of eosinophil function [26] has stood the test of time and has provided a framework for explorations directed at understanding eosinophil function, the roles of eosinophils in both homeostatic/physiologic states and pathophysiologic responses have not been fully elucidated. Many studies involving eosinophil recruitment yield discordant results (for example, see [13–18,49,50]). This may be related to the fact that, despite their unique and distinctive appearance, eosinophils are not a uniform population. Building on the LIAR hypothesis [26], these findings suggest that eosinophils may also be capable of responding to (as well as influencing) the local microenvironment. For example, we showed here that eosinophil (and BAL) cytokine content largely increases in allergen-challenged mice maintained on a high-sucrose diet and that these findings were associated with a diet-dependent increase in airway resistance. The impact of the local microenvironment on eosinophil phenotype was shown clearly by Reichman *et al*. [19] and Sek *et al*. [20] in studies focused on eosinophils associated with tumor tissue and muscle lesions, respectively.

To the best of our knowledge, this is the first study that has addressed the role played by diet in influencing eosinophil phenotype. Significant changes in diet over time can result in metabolic shifts that have a profound influence on systemic cytokine responses [51–53]; these responses may in turn have a discernible impact on the activation state of eosinophils. In this study, it is not clear how eosinophils become cytokine-enriched in response to dietary changes. Preliminary results focused on cytokine contents of lung eosinophils isolated from *IL5*tg mice (i.e., no allergen challenge) provided no significant clarification of this point (Percopo and Rosenberg, unpublished findings).

In summary, we have examined the impact of diet on allergic responses with a focus on the cytokine content of eosinophils recruited to the lungs. Most intriguing, we identified increased airway resistance and changes in BAL and eosinophil cytokine content in mice that gained only minimal weight while maintained on a high-sucrose diet. These results provide a model for further exploration of the unique risks associated with a high-sugar diet and its impact on immunomodulatory and respiratory dysfunction.

## Supporting information

S1 Fig Profiler signals from cytokines detected in BAL of Aa-challenged mice maintained on (A) high-sucrose or (B) high-fat diets normalized to those from Aa-challenged mice maintained on a basic diet. Shown are the results presented from three independent experiments; each point represents n = 5 mice as described in the Methods. Arrows indicate no increase over signal detected in mice on the basic diet (i.e., fold change = 1.0); §p < 0.001 vs. theoretical mean (= 1.0), one-sample t-test.
(TIF)

S2 Fig Serum leptin levels (ng/mL) correlate directly with weight (g) in mice maintained on (A) high-fat and (B) basic diets, but not in mice maintained on a high-sucrose diet (C); n = 48–50 mice per group, p values as shown, simple linear regression.
(TIF)

S3 Fig Profiler signals from cytokines detected in lysates of eosinophils isolated (as shown in Fig 4A) from *Aa*-challenged mice maintained on (**A**) high-sucrose or (**B**) high-fat diets normalized to those from *Aa*-challenged mice maintained on a basic diet. Shown are the results presented from three independent experiments; each point represents n = 5 mice as described in the Methods. Arrows indicate no increase over signal detected in mice on the basic diet (i.e., fold change = 1.0); ^§^*p* < 0.001 *vs*. theoretical mean (= 1.0), one-sample t-test.
(TIF)

S1 Table Contents of diets (Research Diets, New Brunswick, NJ, USA) used to feed mice in this study. The high sucrose diet contains 4–5 times more sucrose than the basic diet, although the total carbohydrate content remains constant. Further details can be found at https://researchdiets.com/.
(PDF)

S1 Appendix (DOCX)

S2 Appendix (XLSX)

S3 Appendix (XLSX)

S4 Appendix (XLSX)

S5 Appendix (XLSX)

S6 Appendix (XLSX)

S7 Appendix (XLSX)
