# Supplementary figures and images for: Impact of controlled high-sucrose and high-fat diets on eosinophil recruitment and cytokine content in allergen-challenged mice
Source: PLoS One. 2021 Aug 12;16(8):e0255997. doi: 10.1371/journal.pone.0255997 (PMC8360545; doi:10.1371/journal.pone.0255997)

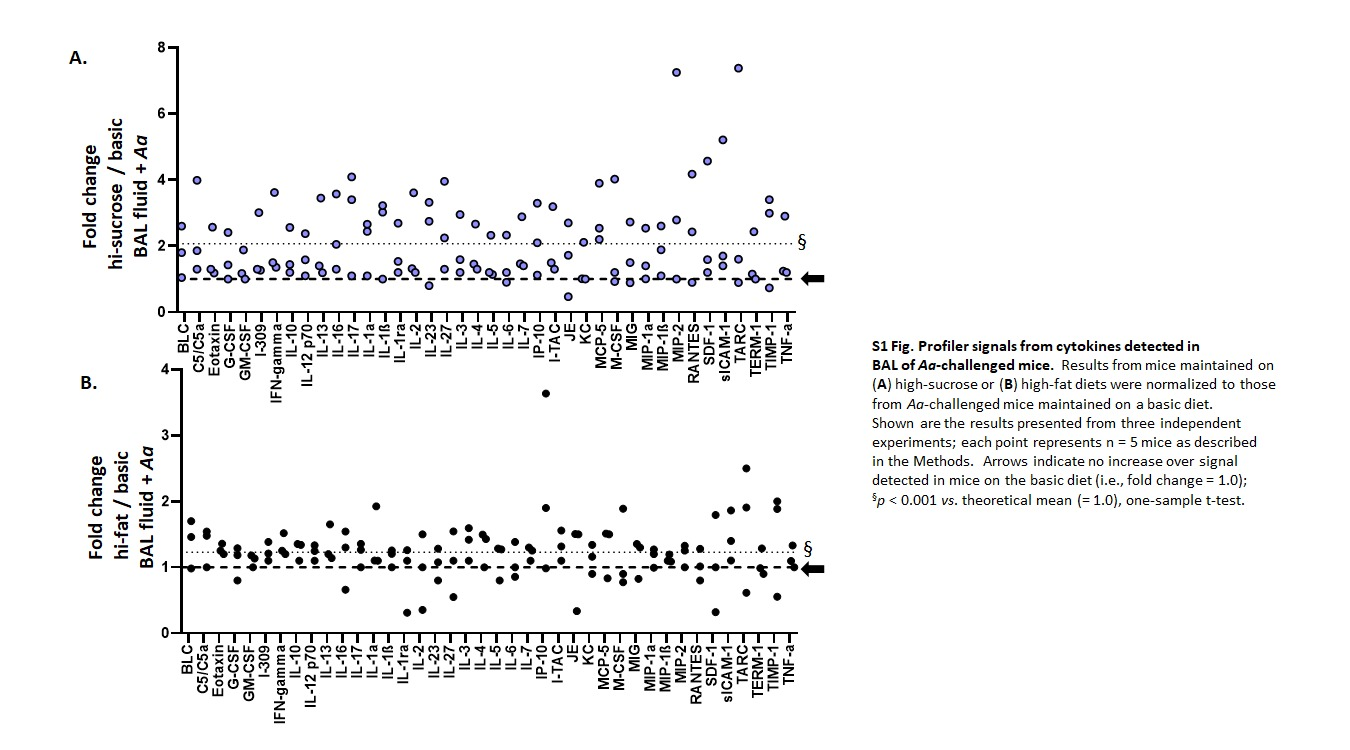

Supplement: S1 Fig — Profiler signals from cytokines detected in BAL of Aa-challenged mice maintained on (A) high-sucrose or (B) high-fat diets normalized to those from Aa-challenged mice maintained on a basic diet. Shown are the results presented from three independent experiments; each point represents n = 5 mice as described in the Methods. Arrows indicate no increase over signal detected in mice on the basic diet (i.e., fold change = 1.0); §p < 0.001 vs. theoretical mean (= 1.0), one-sample t-test. (TIF) [file pone.0255997.s001.tif]

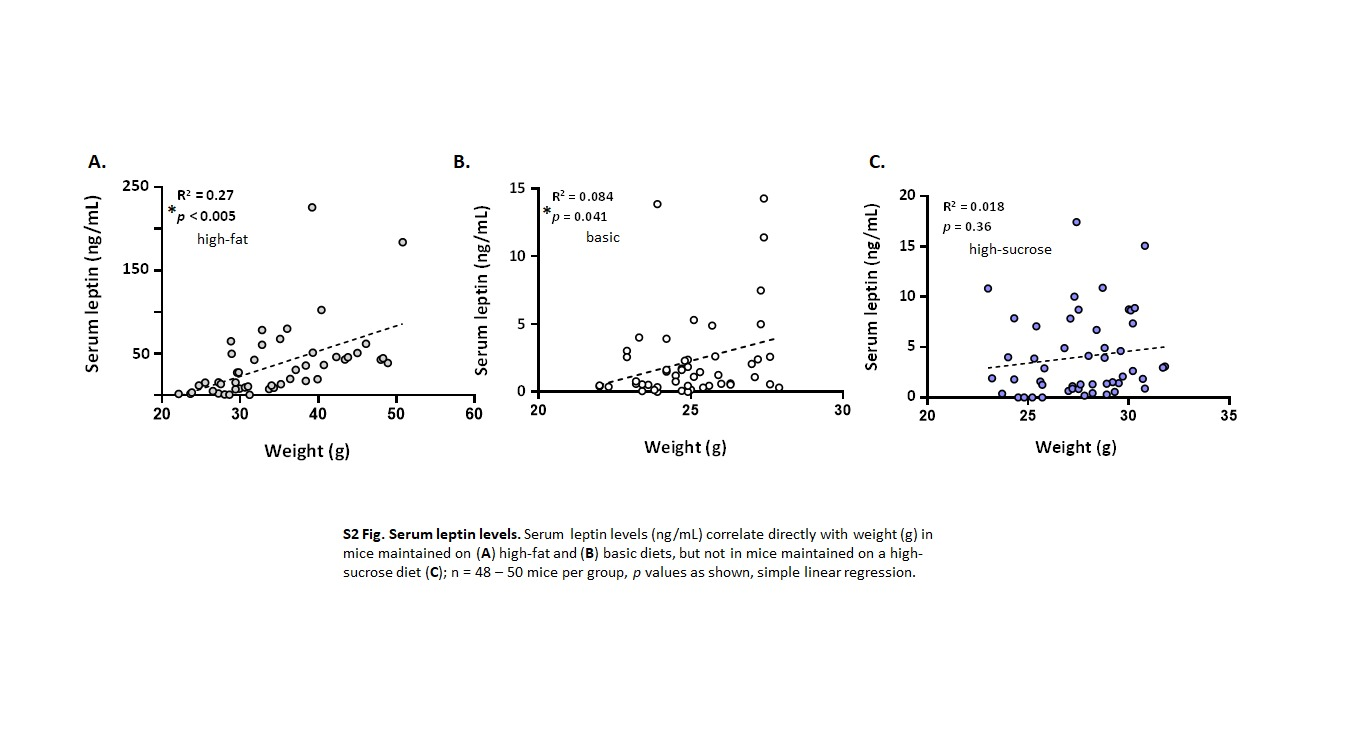

Supplement: S2 Fig — Serum leptin levels (ng/mL) correlate directly with weight (g) in mice maintained on (A) high-fat and (B) basic diets, but not in mice maintained on a high-sucrose diet (C); n = 48–50 mice per group, p values as shown, simple linear regression. (TIF) [file pone.0255997.s002.tif]

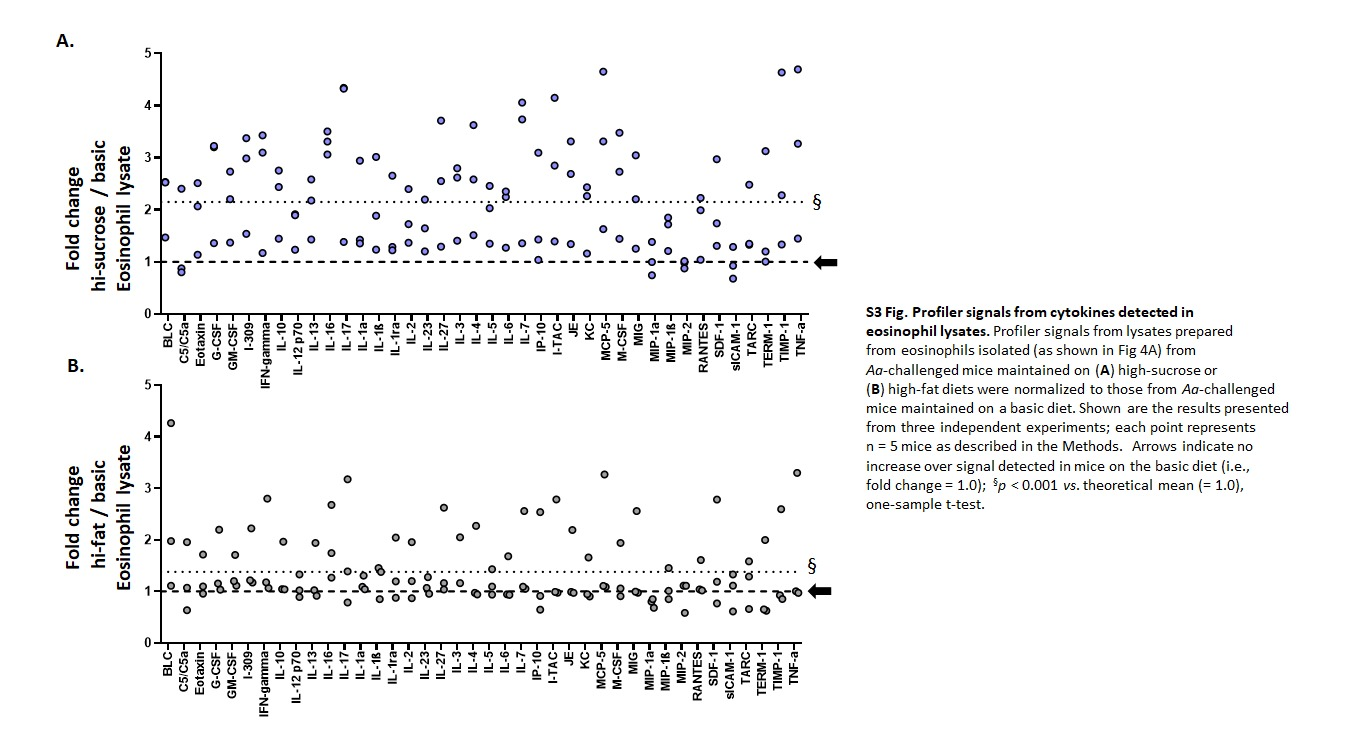

Supplement: S3 Fig — Profiler signals from cytokines detected in lysates of eosinophils isolated (as shown in Fig 4A) from Aa-challenged mice maintained on (A) high-sucrose or (B) high-fat diets normalized to those from Aa-challenged mice maintained on a basic diet. Shown are the results presented from three independent experiments; each point represents n = 5 mice as described in the Methods. Arrows indicate no increase over signal detected in mice on the basic diet (i.e., fold change = 1.0); §p < 0.001 vs. theoretical mean (= 1.0), one-sample t-test. (TIF) [file pone.0255997.s003.tif]
