# Supplementary material for: Impact of controlled high-sucrose and high-fat diets on eosinophil recruitment and cytokine content in allergen-challenged mice
Source: PLoS One. 2021 Aug 12;16(8):e0255997. doi: 10.1371/journal.pone.0255997 (PMC8360545; doi:10.1371/journal.pone.0255997)
Supplement: S1 Table — The high sucrose diet contains 4–5 times more sucrose than the basic diet, although the total carbohydrate content remains constant. Further details can be found at https://researchdiets.com/. (PDF) [file pone.0255997.s004.pdf]

S1 Table. Contents of diets (Research Diets, New Brunswick, NJ, USA) used to feed mice in this study. The high sucrose diet contains 4 – 5 times more sucrose than the basic diet, although the total carbohydrate content remains constant. Further details can be found at https://researchdiets.com/.

**Diet**	**D12450J (basic)**	**D12450B (high-sucrose)**	**D12492 (high-fat)**
**Protein (% of kcal)**	20	20	20
**Fat (% of kcal)**	10	10	60
**Carbohydrate (% of kcal)**	70	70	20
**Sucrose (g/100 g diet)**	6.9	33.6	9.4
**Energy density (kcal/g)**	3.82	3.82	5.21
