# Supplementary material for: Impact of controlled high-sucrose and high-fat diets on eosinophil recruitment and cytokine content in allergen-challenged mice
Source: PLoS One. 2021 Aug 12;16(8):e0255997. doi: 10.1371/journal.pone.0255997 (PMC8360545; doi:10.1371/journal.pone.0255997)
Supplement: S1 Appendix — (DOCX) [file pone.0255997.s005.docx]

**Appendix S1**

**Impact of controlled high-sucrose and high-fat diets on eosinophil recruitment and cytokine content in allergen-challenged mice**

Caroline M. Percopo^1^, Morgan McCullough^2^, Ajinkya R. Limkar^1^, Kirk M. Druey^2^, and Helene F. Rosenberg^1^

^1^Inflammation Immunobiology Section, Laboratory of Allergic Diseases, National Institute of Allergy and Infectious Diseases, National Institutes of Health, Bethesda, Maryland 20892, USA

^2^Lung and Vascular Inflammation Section, Laboratory of Allergic Diseases, National Institute of Allergy and Infectious Diseases, National Institutes of Health, Bethesda, Maryland 20892, USA

**Extended Methods**

**Protocol 1. Preparation of single-cell suspensions from whole lung tissue**

The following is our step-by-step protocol for preparing single-cell suspensions from whole lung tissue. This information can also be found in Percopo CM, Limkar AR, Sek AC, Rosenberg HF. Detection of mouse eosinophils in tissue by flow cytometry and isolation by fluorescence-activated cell sorting (FACS). Method Mol Biol. 2021;2241: 49–58.

**Perfusion medium**

20 mL 0.5 M EDTA

Filter sterilize. Store at 4 °C.

Bring to 980 mL PBS

**Digestion medium (9 ml/lung)**

Prepare fresh each time, use right away

5 mL fetal bovine serum (FBS)

100 uL DNase I stock solution (20 mg/mL)

5 mL collagenase D stock solution (40 mg/mL)

Bring to 100 mL RPMI 1640 (enough for 10 mice)

1. Anesthetize the mouse by isolfluorane inhalation. Sacrifice the mouse by gentle cervical dislocation.
2. With scissors, open the abdomen. Cut the blood vessels that enter the diaphragm. Open the thorax to visualize the lung and the heart.
3. Place the mouse onto an absorbent paper and perfuse the lung by injecting the heart (right ventricle, 12 mL syringe, 27 GA ½ needle) with 8 mL of perfusion medium to remove circulatory blood cells. The lung must appear white after that step.
4. Place the lung into a 6-well plate with 3 ml digestion media.

**Lung digestion**

1. Put the lung in a Petri dish and mince the lung with a straight razor blade. Scoop up lung and transfer to a 15 ml tube.
2. Rinse the Petri dish and blade with 3ml of digestion media from 6-well plate. Then rinse wells and plate with 3 mls of fresh digestion media and transfer to 15 ml tube containing minced tissue.
3. Incubate at 37 °C on a turning wheel for a total time of 90 min. After 45 min of incubation (half time point), add 3 mL of fresh digestion medium. Pipet up and down.
4. Place the conical tube at room temperature.
5. Add 0.5 M cold EDTA to each tube to a final concentration of 10 mM EDTA (50-fold dilution; 180 µl in 9 ml). Pipette up and down.
6. Incubate 5 min. Pipette up and down.
7. Set a 70-micron cell strainer on a 50 mL conical tube. Pour cell suspension to the filter. Add 5 mL cold Wuerzburg buffer (MACS buffer replaces Wuerzburg buffer if a MACS purification follows this protocol) to the empty conical tube to wash out any residual cells. Add the volume to the filter.
8. Centrifuge 5 min at 1200rpm at room temperature to pellet cells.
9. Aspirate supernatant. Add 1–2 mL of room temperature ACK lysis buffer. Incubate 2 min. Add 10 mL of Wuerzburg/MACS buffer. Mix the cell suspension. Transfer to a 15 mL conical tube.
10. Centrifuge 5 min 1200 rpm at room temperature to pellet the cells.
11. Aspirate the supernatant, resuspend pellet in 10 mL HBSS buffer. (If a visible clump has formed, filter the cells.)
12. Enumerate the cells.
13. Staining for flow cytometry or FACS: Resuspend cells at 1x10^6^ cells/100 μl sample in PBS and follow protocol as desired; see citation listed above for futher information on isolating eosinophils.

**Buffers**

(keep all on ice)

**Wuerzburg buffer (WB)**

Prepare without the DNase I. Add the enzyme the day of use.

1.5 g BSA

5 mL 0.5 M EDTA pH 8.0

Bring to 500 mL with PBS

***Add 100 uL of DNase I stock solution (20 mg/mL) per 100ml WB (enough for 5 mice)***

**DNase I stock solution (20 mg/mL)**

100 mg DNase I grade II (Roche)

solution (cf. supra)

Bring to 5 mL with glycerol/CaCl_2_/Na-acetate (see below)

Filter sterilize.

Prepare 250 µL aliquots. Store at -20 °C

**Glycerol/CaCl_2_/Na-acetate solution**

50 µL CaCl_2_ 1M

83 µL Na-acetate 3M

25 mL glycerol

Bring to 50 mL with deionized water

**Collagenase D stock solution (40 mg/mL)** lyophilized at 4^o^C.

2.5 g collagenase D (Roche)

Bring to 62.5 mL with PBS

Filter sterilize, if cells are to cultured.

Prepare at 1.5 mL aliquots.

Store at -20 °C

**Protocol 2. Cytokine Profiling**

The following is our step-by-step protocol used for performing proteome profiling experiments.

Profiling of BAL fluid and eosinophil lysates was performed using the Mouse Cytokine Array Panel A (catalog no. ARY006, R & D Systems, Minneapolis, MN) that was adjusted by the manufacturer to use IR Dye 800CW and detection on a LI-COR imaging system. The manufacturer’s instructions (see https://www.rndsystems.com/products/proteome-profiler-mouse-cytokine-array-kit-panel-a_ary006) were followed as previously described [20, 28].

1. Instructions provided by the manufacturer for use the profiler arrays for LI-COR detection were followed throughout.
2. Combined samples (total 300 µg eosinophil lysate protein combined from equal aliquots from n = 5 mice or 1 mL BAL fluid, 0.2 mL per mouse combined from n = 5 mice) were incubated with the Mouse Cytokine Array Panel A detection antibody cocktail for one hour at room temperature.
3. After removal of the stamped identification number on each membrane, samples (combined BAL fluids or lysates that had been incubated with the aforementioned antibody cocktail) were then added to pre-wetted membranes embedded with capture antibodies that were provided in the kit.
4. The sample and membrane were incubated overnight at 4^o^C on a rocking platform.
5. Membranes were then washed as per the manufacturer’s instructions.
6. Membranes incubated for 30 minutes with a 1:2000 dilution of IRDye 800CW Streptavidin (catalog number 926-32230, LI-COR Biosciences, Lincoln, NE) at room temperature on a rocking platform (2 mL per well for a total of 4 mL in a 4-well plate) that was protected from light.
7. Membranes were then subjected to a final washing step. Excess buffer was removed by blotting the membrane onto absorbent paper.
8. While transporting the membrane to the scanner, the membranes should remain in each well in a small amount of wash buffer to keep them from drying out.
9. Data were collected using an Odyssey CLx imaging system (LI-COR). Each membrane should be placed face down and upside down on the scanner. Scanning parameters include channels, 800; resolution, 84 µM; quality, medium (adjust as necessary); focus offset, 0.0 mm; intensity, 5 (adjust as necessary); analysis mode, grid array.

**Protocol 3. Airway resistance in response to methacholine.**

The following is our step-by-step protocol for examining methacholine-dependent increases in airway resistance in mice using the flexiVent system. The flexiVent User manual can be obtained from <https://www.scireq.com/flexivent/>.

**Preparation.**

One day before the planned experiments, a dose-response determination is performed. Ensure that you are in the desired database and create a new study using the flexiWare software under “STUDY DEFINITION & PLANNING” as described in the flexiWare Software manual.

1. If needed or if starting a new study, calibrate the flexiVent Pcyl channel, Pao channel, and Aeroneb as described in the flexiVent User manual.

**Running the experiment.**

1. Prepare dilutions of methacholine in phosphate buffered saline (PBS). Also include a PBS only tube for the 0 mg/mL methacholine dose.
2. Place the Aeroneb onto Nebulizer Y-tubing Assembly and connect to the flexiVent apparatus.
3. Plug Nebulizer cable into Aeroneb and load ~500 μL of PBS into the Aeroneb.
4. Start flexiVent.
5. Start flexiWare software.
6. Wait for status on flexiVent screen to read “Idle” and Click on “EXPERIMENTATION SESSION”.
7. Select the study that you want to run and click “Ok”.
8. Select the experiment template that you want to run (FlexiVent FX-Mouse AN) and click “Ok”.
9. Enter operator’s name or initials and click “Ok”.
10. Confirm that the module is attached and click “Ok”.
11. Weigh mouse and enter the weight in the current weight box in the flexiWare software and click “Ok”.
12. Click “Skip warm-up” in the flexiVent Auto-Calibration window.
13. Click “Ok” when prompted to Prime Aeroneb.
14. Gently wipe the condensation from inside of the underside of the Aeroneb and return it to the Y-tube assembly.
15. Click “Restore” on the Channel selection screen for calibrations. If any of the boxes are checked those channels will have to be recalibrated before continuing (see flexiVent User Manual for details about calibrations). Or, if recalibration is desired, one or all of the boxes can be checked and calibration(s) performed.
16. Observe calibration values given on the Calibration values screen. If all of the values are within the limits listed then click “Next”. If not, recalibration is required.
17. Click “Finish” on the Final Step Calibration Complete screen.
18. Connect a pink 19-gauge cannula to the Y-tubing and perform tube calibration as follows: Click “Next” on the welcome screen → verify that all four perturbation boxes are checked on the Perturbation Selection screen and click “Next” → completely block the end of the cannula either with your thumb/finger or using one of the cannula blocks and click “Next” (the flexiVent will now perform the closed tube calibration – do not remove finger/block from cannula during this time) → unblock the cannula and click “Next” on the Open Preparation screen (the flexiVent will now perform the open tube calibration) → verify that all four tests have blue arrows (↕) shown in front of their names and click “Finish” (if any one of the tests have a red triangle then click “Start Over” and repeat the tube calibration as above). EVERY new tube must be calibrated for each mouse before use. Also, note that it is only for the first mouse that you will have to prime the nebulizer and do the initial restoration of the instrument calibrations. All the remaining mice will simple be added by right clicking on the Site 1 window → click “Edit Site Assignment” → highlight desired mouse on list → click the “→” arrow → click “Ok” → enter current weigh and click “Ok” → click “Ok” (you will then again be stepped through the cannula/tube calibration).
19. DO NOT click “Yes” or “No” when asked if you wish to start the default ventilation pattern (the default ventilation pattern should be started just as a mouse is being connected to the flexiVent).
20. Anesthetize mouse using Ketamine/Xylazine as per standard procedure.
21. Perform tracheostomy and insert a 19 gauge cannula (pinch skin above trachea and cut to reveal a circular shaped opening → gently separate salivary glands using forceps → gently separate muscle surrounding trachea using forceps → dissect out trachea using one curved and one straight forceps and insert a strand of 4-0 silk beneath the trachea → make a small nick in the trachea between the cricoid cartilages and insert the cannula until the tip reaches the apex of the chest → tightly secure the cannula with the 4-0 silk using a surgeon’s knot).
22. Connect mouse to flexiVent and the start default ventilation by clicking “Yes”
23. Administer Vecuronium (paralytic) and record the time.
24. Wait EXACTLY 5 minutes after connecting the mouse and record the Scan Start time and perform a manual “Deep Inflation” (to check for potential leaks).
25. Wait for 10 default ventilation pulses (~3 sec) and perform a manual “PVs-P” (to check for spontaneous breathing activity).
26. Wait for 10 default ventilation pulses (~3 sec) and initiate the “Mouse Inhaled Dose Response” script. The script will begin with two automatic Deep Inflation maneuvers.
27. When prompted, enter “0mg/mL” for the dose concentration and “15%” for the duty cycle and click “Ok”. The script will again run twelve repeats of each of the preselected maneuvers for this dose.
28. Click “Ok” when asked if you wish to do another dose. The script will again do two automatic Deep Inflation maneuvers.
29. Repeat the two steps above for each additional dose, entering the new concentration for each dose when prompted, and check that dose off on the Methacholine Dose Checklist sheet. Typical doses/concentration include: Baseline (BL), 0mg/mL, 6.25/mL, 12.5 mg/mL, 25 mg/mL, 50 mg/mL, and 75 mg/mL). Also, don’t forget to remove the solution for the previous dose from the Aeroneb and replace it with the next dose. Do this carefully using a Kimwipe. To save time, the doses (liquid) can be removed and added during the default ventilations between the two Deep Inflation steps (but never while the Deep Inflation maneuvers are being done since readings are being taken at those times).
30. After the last dose has been run, click “Cancel” when asked if you wish to do another dose.
31. Right-click on the Site window and choose “Edit” then “Change Subject”
32. Remove the mouse from Site 1 by clicking the “←” arrow and clicking “Ok”
33. Stop the default ventilation pattern by clicking “Ok” and “Ok” when asked if the experiment was terminal.
34. The Aeroneb and Y-tubing assembly should be thoroughly cleaned between mice as follows: unplug cable from Aeroneb → remove Aeroneb from Y-tubing assemble and rinse the top and bottom of the screen with water, gently drying with a Kimwipe → rinse all ports of Y-tubing with water and blow dry with house air until TOTALLY dry inside and out → reassemble Aeroneb and Y-tubing assembly and reconnect to flexiVent → reconnect cable to Aeroneb → add PBS to Aeroneb for 0 mg/mL dose.
35. Add a new mouse to Site 1 as done previously (by right-clicking the Site 1 window) and repeat all of the above steps for each mouse (to save time spent waiting for the anesthetic to take effect, mice can be anesthetized during the last methacholine dose of the previous mouse – this however should not be tried with mice that are very sick).
36. After the last mouse, end the experiment by closing the program (red X at top right of screen).
37. The program will now step you through the washing procedure. For most procedures a 5-minute water wash and soak will be sufficient. Some procedures may require a weak detergent solution first be used before the water soak. IF INFECTED MICE WERE DONE → first rinse the system with isopropanol followed by the water rinse and soak.
38. Before beginning the wash→ remove the Aeroneb and the Aeroneb Y-tube assembly from the flexiVent and clean them as done previously between mice (allow the Aeroneb to dry laying on its side) → connect one end of a short piece of tubing to the outlet port of the flexiVent and place the other end in an empty beaker (used as a drain tube) → fill a 60cc syringe with wash solution → attach another short piece of tubing to the end of the syringe and then plug it into the far right port in the front of the flexiVent where the Y-tube assemble would normally be attached.
39. Follow the on-screen prompts to complete the washing procedure (all of the wash solutions must be pushed through the flexiVent during the 5-minute soak and before the start of the drying step) (some air should also be pushed through immediately before the end of the 5-minute soak until liquid can no longer be seen coming from the outlet port).
40. Disconnect the syringe and syringe tubing from the front of the flexiVent and connect the Mouse Mechanics Scans Y-tube assembly (this is not the Y-tube assemble used for the Aeroneb) → block the end of the Y-tube assembly.
41. Follow the on-screen prompts to complete the 10-minute drying procedure.
42. After the drying step is complete, disconnect the Y-tube assembly as well as the tubing going from the outlet port to the beaker and wait for the program to exit.
43. Turn off the flexiVent.
